# Review of State-of-the-Art FPGA Applications in IoT Networks

**DOI:** 10.3390/s22197496

**Published:** 2022-10-02

**Authors:** Alexander Magyari, Yuhua Chen

**Affiliations:** Department of Electrical and Computer Engineering, University of Houston, Houston, TX 77204, USA

**Keywords:** FPGA, IoT, networks, NIC, high perforamce, low power, ASIC

## Abstract

Modern networks used for integrating custom Internet of Things (IoT) systems and devices have restrictions and requirements unique to their individual applications. These application specific demands require custom designed hardware to maximize throughput, security and data integrity whilst minimizing latency, power consumption, and form factor. Within this paper, we describe current, state-of-the-art works that utilize FPGAs for IoT network developments. We analyze two categories of works: those that prioritize reducing power consumption, and those that prioritize networking features. Further, we describe how future works can improve upon these designs and therefore improve the efficiency of resource-constrained IoT networks.

## 1. Introduction

The Internet of Things (IoT) is a vast system of integrated devices and services that include data acquisition via sensors, electromechanical machines, software processing, and features for human interaction. These devices and services are connected via the Internet, where a system of devices often interact on a local, customized network to fulfill a specialized purpose. As the needs for these custom network evolve, so must the hardware and software that supports them. Current areas requiring areas for network improvement include:

- **Increasing Throughput:** The Ethernet road map suggests that future networking switch interfaces will realize speeds of 800 Gigabits per second (Gbps) to 1.6 Terabits per second (Tbps) after 2022 [1]. For these rates to be obtained, high processing speeds will be required of packet classification and parsing algorithms.
- **Decreasing Power Consumption:** Dennard Scaling, which states that the power density of transistors stays roughly the same as transistors get smaller (especially in accordance with Moore’s Law), has come to an end with field effect transistor (FET) technologies. It is now accepted that the power density rapidly increases within a chip as manufacturing processes continue to decrease in size [2]. The end of Dennard Scaling leads to requirements for power gating, often referred to as dark silicon, to prevent catastrophic failure in multicore systems [3].
- **Decreasing Form Factor:** Similarly to the end of Dennard Scaling, Moore’s Law, the acceptance that transistor size will decrease by half roughly every 18 months, is slowing down [4]. Lowering the area required for an integrated circuit can reduce the cost of production, reduce power consumption, and increase potential for implementation in devices that demand a small form factor [4].
- **Reducing Latency and Jitter:** Latency refers to the delays in packet processing, and jitter refers to the variation in latency over some sample of time. Predictable latency and reduced jitter allow for more efficient networking algorithms, thereby increasing performance [5]. Latency and jitter are often a severe bottleneck in mobile and wireless applications.
- **Recovery of Dropped Packets:** IoT networks face the unique challenge that typical networks supported by TCP/IP do not: edge nodes that go in and out of range and nodes that go into sleep mode. This unpredictable behavior can lead to dropped packets and unrecoverable data [6,7,8]. Further, dropped packets can lead to violations of sequential dependencies, significantly increasing latency or introducing hanging conditions [9].
- **Improving Network Security:** IoT networks are often the target of cyber-attacks, as many networks reduce safety measures to improve power efficiency [10]. These vulnerable edge nodes offer an entry-point to broader networks, and can be used to compromise valuable data [11,12].

This article will focus on efforts to improve upon the aforementioned networking topics by utilizing Field Programmable Gate Arrays (FPGAs). As the works reviewed in this paper will reveal, FPGAs can be used at many corners of application areas for IoT networking, potentially filling the gap between Application-Specific Integrated Circuits (ASICs) and embedded processors, depending on the applicaiton.

This review will have importance to future designers who seek to further expand the abilities of reconfigurable hardware in the networking realm, including those interested in Single Event Effect (SEE) correction [13,14] and those interested in applications for partial reconfiguration [15,16]. Future research into FPGA networking capabilitiescan be expedited by reviewing the state-of-the-art works described within this paper. Additionally, this work will serve as a summary of current milestones that must be exceeded for FPGAs to continue to stay relevant to IoT networking.

Our review is organized as follows: in Section 2, we give a brief history of advances in IoT networking and describe the fundamentals of FPGAs. In Section 3, we cover current advances in high-performance IoT networks. In Section 4, we discuss recent works that approach IoT networks with power-saving abilities in mind. In Section 5, we discuss some of the commonalities found within the reviewed works and why they are important to future iterations. Finally, we conclude the paper in Section 6, where we also discuss when it would be beneficial to use FPGAs over ASICs in an IoT system design.

## 2. Background

This section is intended to give the reader context about previous advancements in IoT networking and a high-level background on FPGAs. While the papers reviewed in this article serve very specific purposes and may not be relevant for some applications, the IoT networking additions described within this section provide benefits that serve a broad variety of IoT networks.

### 2.1. Historical IoT Network Technologies

Classic networking protocols, such as IPv6, while having the potential for utilization with IoT networks, are often not the best choice as they are not optimized for the demands of such networks. There are multiple reasons behind this, although these reasons can be summarized as having too much unnecessary overhead, increasing power demands and network latency. For example. IPv6 requires all compatible networks to support a Maximum Transmission Unit of 1280 bytes [17,18], which is often unnecessary to transfer the data on IoT networks. Further, IPv6 headers include data not pertinent to IoT networks, such as flow labels and traffic class. For purposes related to IoT, these headers and otherwise unnecessary data transfers should be either reduced or eliminated entirely.

One of the first protocols designed for IoT networks was Message Queuing Telemetry Transport (MQTT), an application-layer based protocol that was started by IBM. MQTT was specifically designed for nodes that operate in remote locations and have resource constraints and limited bandwidth. MQTT typically operates over TCP/IP, relies on the publisher/subscriber relationship, and has a fixed header size of only 2 bytes [19,20,21].

One other popular IoT networking protocol is IPv6 over Low-Power Wireless Area Networks (6LoWPAN) and is a direct answer to the IPv6 issues outlines above [22]. 6LoWPAN allowed for networks to get around the issues of unnecessary overhead associated with IPv6, allowing for header compression from 40 bytes to 7 bytes. 6LoWPAN also introduces link layer fragmentation, which veils the real MTU size, allowing for smaller MTUs with the appearance of 1280 byte MTU support [23,24].

One other major networking protocol currently utilized on IoT networks is the Constrained Application Protocol (CoAP) [25], which is a web-based transfer protocol that is specifically designed for resource constrained devices, such as those on IoT networks. It is built upon the UDP protocol and focuses on the common representational state transfer (REST) paradigm, similar to Hypertext Transfer Protocol (HTTP) [26,27].

### 2.2. Field Programmable Gate Arrays

Field Programmable Gate Arrays (FPGAs) combine the scalable parallel processing capability of an Application Specific Integrated Circuit (ASIC) and the reprogrammability of a conventional processor. FPGAs are typically designed using Hardware Description Language (HDL), which is a unique language that describes precise hardware functionality. By writing various forms of HDL, digital designers can implement a wide array of hardware functions that can operate similarly to an ASIC.

The primary difference between an FPGA and an ASIC is that an ASIC has optimized hardware and logic gates uniquely implemented for its application, whereas an FPGA combines the usage of look up tables (LUTs) and configurable routing through the utilization of programmable static random access memory (SRAM) cells to implement logic functions. The primary benefit of the FPGA relative to the ASIC is the reprogrammability of the FPGA. If a hardware issue is detected, or an optimization is made to the hardware design, the FPGA can be reprogrammed without the physical device having to be redesigned. Contrarily, the ASIC will need to be entirely redesigned, fabricated, and distributed. The downside of the FPGA hardware compared to the ASIC is that the FPGA will have a lower maximum operating frequency, higher real-estate requirements, and will often times have non-optimal power usage [28,29,30,31].

FPGAs can be useful at nearly every layer within the OSI model and have been utilized as networking components in the industry. For example, the Microsoft Catapult System [32,33] and Amazon AQUA [34] utilize FPGAs with direct network access for their data centers. Furthermore, works such as FDGLIb are utilities that streamline FPGA capabilities within datacenters, such as those from Microsoft and Amazon [35]. We encourage the reader to refer to [28,30] for more information regarding the architecture and applications of FPGAs. 

## 3. High Performance Networks

We define high-performance IoT networks as those that do not have a focus on energy saving techniques. Rather, high performance networks focus on other areas of networking, such as maximizing throughput, increasing resistance to radiation, or minimizing latency for devices such as robotics that require a near-immediate response to a remote input. As will be shown in the following subsections, many high-performance systems are often hard-wired through Ethernet or optical cabling, and have access to a constant power source.

Within this section, we discuss recent advances in high-performance IoT networks and list the benefits, as well as the shortcomings, of each work. We then describe future areas for improvement and describe how FPGAs can be used to meet these requirements.

### 3.1. Radiation Tolerant Networking

FPGAs are a promising solution for unique networking requirements, especially those in high-radiation environments, such as applications in space, nuclear power plants, and in unique lab-based research areas. Unique to these environments come the troubles of bit errors caused by single-event effects (SEE). Historically, answers to SEEs have historically come in the form of anti-fuse FPGAs, which allow for one-time-only programming (OTP) [36,37,38,39].

Unfortunately, OTP-based FPGAs lose the primary-benefit of FPGAs: the ability for reconfiguration in the field. Space applications have an especially significant need for the reconfiguration that SRAM-based FPGAs provide. If a bug or disturbance caused an OTP FPGA to malfunction while in space or an otherwise inaccessible area, providing a replacement would prove to be unattainable. Therefore, there is a need for digital programming methods that allow for SRAM based FPGAs to self-correct any bit errors that accumulate due to SEEs.

A multi-gigabit serial link was developed that provides a novel approach to correcting SEEs in both data transmission and localized data bits [13]. Regarding network transmissions, this work relies on configurable amounts of Reed-Solomon encoding (RSE), where the amount of encoding has an inverse-proportional relationship with the provided network throughput. RSE is an error correction method that operates on a block of data, such as an Ethernet packet, and treats each block as a set of finite-field elements [40]. Unlike other bit-correction methods, RSE can detect and correct multiple symbol errors. Within the multi-gigabit serial link, the configuration scheme provides a maximum throughput of 5.8 Gbps with no RSE, or up to a maximum correction capability of six 4-bit symbols of consecutive corrupted bits at 3.3 Gbps.

Further, the implementation of this design includes a scrubber, which allows for the correction of localized bit errors within the design. Through the incorporation of the Xilinx internal configuration access port (ICAP), the radiation-tolerant scrubber scans a list of triplets of configuration frames. The scrubber assumes the triplets to be identical; if differences are detected amongst the triplets, the error is resolved through majority-voting. The update to the SRAM is completed via partial reconfiguration of the frames, with scrubbing of the entire device completed every second.

While this work does provide an adaptation for RSE that has a reconfigurable throughput, the scrubber has some areas for improvement. The use of triplets requires tripled memory, which results in increased power usage as well as an increased footprint. In instances of large SEEs, there exists a non-significant probability in which two or more bits within a triplet are erroneously flipped, causing the scrubber to overlook errors. This build-up of uncorrected errors can cause a design to fail completely. Therefore, this implementation of bit-correction may only be useful in applications with low to moderate radiation exposure.

An alternative option for bit-errors within configuration data exists [14]. This work utilizes dynamic-reconfiguration, similar to the previously mentioned work. However, this option reduces the memory requirement by using Cyclic Redundancy Checks (CRC) [41] against the configuration bit stream. Through the use of CRCs and radiation-tolerant memory, this adaptation contains parameters for bit-error corrections with a single copy of the configuration, rather than two. Additionally, assuming that the memory where the configuration is stored is entirely radiation-resistant, this work is not exposed to the threat of multiple bit-errors with the potential of uncaught corrections. Combined with the RSE of the previously discussed networking implementation, this approach could prove to be a successful adaptation of networking in high radiation environments.

### 3.2. Minimizing Latency

Networking latency, often measured in the time it takes to send one packet from a start node to an end node and back, is essential for networks in which there is some form of remote control. This is a typical need of robotic-centric systems or any network with a node that has a remotely controlled actuator [42,43,44]. The difficulty in mitigating latency within networks is that methods for managing latency are often application specific, meaning that each networking application will require its own approach to reducing network latency.

Classic approaches to networking, such as non-specific TCP/IP stacks, are non-ideal for low-latency applications. Reasons behind this include TCP handshaking, Head-of-line blocking in lossy networks, and unnecessary ACK/NAK transmissions.

An example of an application-specific, low latency approach that often requires continuous improvements in latency reduction is High Frequency Trading (HFT). HFT requires ultra-low latency to beat competitors for buying optimally-priced stocks [45,46,47]. Recently, an adaptation of TCP/UDP stack for HFT sees end-to-end latency of less than 500 ns [48]. The architecture for this application relies on a unique implementation of classic TCP/IP networking, in which a financial protocol encoder/decoder was integrated in between the networking layer receiver and transmitter. This effectively moves the application layer to a direct hardware interface with the networking layer, resulting in the significant reduction of latency. The primary issue with applying this methodology to future works is that this approach is extremely application specific, and may not directly apply to future advances in IoT.

However, this work does demonstrate how integrating an FPGA into a custom TCP/IP network can provide substantial reductions in networking latency. Kao et al. utilized the availability of FPGAs with an integrated network and physical layer for Ethernet transport, allowing for focus on latency reduction through digital systems, rather than spending time and effort on board design. They were able to integrate a Physical Coding Sublayer (PCS) core from Xilinx, further streamlining the process of their design. Similar approaches that rely on FPGAs integrated with pre-fabricated hardware, in which the application layer is directly integrated between the receiver and transmitter, with modifications of the application interface, may prove useful in future works.

A less application-specific approach for latency reduction implements a method for reorganizing data packets before processing them [49]. Specifically, this work was applied to auditory and visual data, in which packet ordering is essential. The data payload is separated from the packet header, and is stored in localized DRAM, while the metadata from the header is sent to reordering buffers. The metadata is input into a FIFO buffer with a modified output scheme. As opposed to the traditional access method where the first data input is the first data output, a middle stage re-organizes the FIFO. The output data of the first stage is accessed by the reordering unit via its sequence number, which is derived from the metadata. The address of the metadata with the lowest sequence number in the reorganization unit is passed to the DRAM, where the payload with the corresponding header is read out into an output FIFO, thereby storing the data in order.

Another approach reduces audio/visual latency transmission to less than 5 ms, but over distance up to 300 km [50]. This FPGA implementation combines a Tiny Codec (TICO) [51] with a Gigabit Ethernet interface to achieve these results. Prior to Ethernet frame transmission, the frames are filtered based on the hardware’s configuration for transmission/reception of packets. Further, to allow for an adaptation to the rate of incoming data, the receiver utilizes a rendering clock adjustment. The input clock of the receiver is driven by the incoming network data, which is passed to a PLL, which stabilizes the rendered picture on the receiver’s end. This approach, while utilizing a hard-wired Ethernet connection, could be applied to wireless video applications for use in drone-based systems.

Another common approach for integrating nodes onto the IoT network is through the integration of 6LoWPAN. Unique to 6LoWPAN is an adaptive layer that allows for header compression and link layer fragmentation. This results in a significant a reduction of power, as typical IPv6 requires fixed 40-byte headers that are often unnecessary large in IoT applications. IPv6 also requires all IPv6 compliant networks to support Maximum Transmission Units (MTUs) of 1280 bytes, which far exceeds the requirements of IoT networks. 6LoWPAN circumvents this through the use of Link Layer fragmentation [22].

A recent work moves the traditionally wireless 6LoWPAN protocol to Optical Wireless Communications (OWC) systems [52]. This OWC system integrates an IoT gateway and an IoT edge node, where the node relies on 6LoWPAN. This implementation sees delay reductions of 5% and throughput gains of 20% when compared against traditional IPv6. An FPGA was utilized as the mediator between the wireless node and the optical network, where incoming wireless packets were stored in an Rx FIFO to be transmitted over the optical cable. The ability to move IoT edge node traffic over an optical network can prove significant in that it will allow for both reduction in latency and improvements in throughput.

### 3.3. Maximizing Throughput

Many IoT networks require high amounts of data throughput, especially those that integrate high resolution video feeds such as applications involving drones [53,54,55,56]. FPGA designers have addressed these challenge of increasing networking throughput by implementing FPGAs as Network Interface Cards (NICs); it is known that FPGA’s capabilities for parallel processing can be utilized to create entire TCP/IP stacks. Historically, FPGA-based networks have been able to achieve throughput in the 10 Gbps range [57,58,59,60]. Despite this, the demand for more throughput is increasing with the scaling of data centers. It remains uncertain how much throughput can be achieved on FPGAs, although the current ceiling hovers around 100 Gbps.

Limago [61], the first FPGA-based, open source, 100 Gbps TCP/IP stack, was developed and released in late 2019. Limago is developed via Xilinx High-Level Synthesis (HLS) and is an expansion of a previous design that reached 10 Gbps speeds [57]. From the aforementioned 10 Gbps design, Limago inherited the scalability for total amount of concurrent connections as well as design control flow and congestion avoidance. Limago expands upon this previous design by expanding the data path eight-fold and doubling the operating frequency, which requires several non-trivial modifications such as pipe lining.

Further, Limago implements a TCP Window Scaling option, which is a direct answer to the Long Fat Pipe Network (LFN). A LFN arises when a network becomes “long” (high latency) and “fat” (high bandwidth), where the “longness” and “fatness” combine to make the TCP/IP connection inefficient. By utilizing Window Scaling, Limago is able to reduce the high bandwidth requirement, in turn making the network more efficient as sequential packets spend less time waiting for clients to acknowledge the reception of previously sent packets.

Limago doubled the clock frequency of the original 10 Gbps stack, resulting in significantly less time for signals to propagate. This leads to an increase in overall logic resource usage, as complex functions must be spread over multiple clock cycles. Moreover, with packet sizes starting at 64 bytes, Limago requires a processing rate of 148.8 million packets per second to reach the line rate of 100 Gbps. This significantly large bandwidth gives rise to the LFN that was not necessarily present in the 10 Gbps stack.

Another 100 Gbps FPGA NIC implementation, EasyNet [62], released in 2021, is an open source networking stack implemented on the HLS Xilinx Vitis platform. Vitis Unified Software Platform is an integrated development environment (IDE) that allows users to rapidly deploy applications on multiple Xilinx platforms, including embedded systems and FPGAs [63]. The EasyNet platform was developed to reduce the effort required for distributed networking applications on an FPGA. EasyNet seeks to shift away from the classic point-to-point serial links that rely on fixed networking models [64,65], as they are not viable for large data centers or IoT nodes. The authors in [62] implement a full TCP/IP stack through HLS to complete these goals. HLS is one layer of abstraction removed from HDL and allows the designer to implement hardware functions in adaptations of C and other common programming languages [66]. The developers of EasyNet imposed five constraints to guide their design:

1. The network stack must be generic enough to support a variety of applications.
2. The infrastructure must be abstracted away from the developer for ease of use and must be usable via HLS.
3. The Vitis integration should not decrease network performance.
4. The EasyNet communication primitives should have a low hardware footprint whilst maintaining a high network throughput.
5. The primitives should be callable as a function in an HLS library.

The EasyNet infrastructure utilizes three different kernels, a CMAC kernel, a networking Kernel, and a user Kernel, each with their own customizable properties that can be applied to unique networking constraints.

The final FPGA NIC implementation, Corundum [67] is an open-source network interface that is proven to have reached the near-100 Gbps mark at 94 Gbps. Released in 2020 and, unlike the previous two designs, EasyNet and Limago, Corundum is implemented in Verilog HDL, rather than being based on HLS. Corundum was developed with three primary objectives in mind:

1. The TCP/IP interface that is to be designed must achieve modern line rates of 100 Gbps.
2. The design must be portable, and therefore configurable, between a variety of network setups.
3. The design must be open source to provide availability to multiple projects.

Forencich et al. sought to reach these targets with two primary developments: an interface developed via HDL on an FPGA, and a driver that can communicate with the HDL design.

The architecture of Corundum is separated into three core sections: the top level module, the interface module, and the port module. The top level module contains the required connecting blocks for the underlying modules, including the Peripheral Component Interconnect Express (PCIe) IP block, the AXI Lite master IP block, the Direct Memory Access (DMA) interface, the Precise Time Protocol (PTP) hardware clock, and the required Ethernet components including the MAC and physical interface.

Further, the top level module includes one or more interface module instances, where each instance of an interface corresponds to an operating system level network interface, such as *eth0*. Within each interface module is queue management logic and completion handling logic. The finite state machine within the queue contains all events within the TCP/IP lifecycle, including *transmit, transmit completion, receive, receive completion*, and other events.

It is clear from the presentation in this subsection that FPGA networking implementations can keep up even with high performing ASICs, and are not limited to low-throughput networks. The benefit from the FPGA implementation comes with their reconfigurability, with each of the aforementioned approaches having their own scale of customization that can be applied depending on the requirements of a network.

### 3.4. Future Directions

Many of the applications listed in this subsection focus purely on performance, with little emphasis on power saving or wireless applications. To have a broader impact on IoT applications, future works should implement the aforementioned works but with more focus on power-saving features. This is especially necessary for IoT nodes that do not have access to a stable power source, and instead rely on battery or solar power [68,69,70].

Further, as with drones, there exists a necessity for high-throughput *wireless* implementations of configurable networking configurations. The three high-throughput implementations presented within this section of the review all require hard-wired Ethernet connections, and would therefore be incompatible with drones or other non-stationary edge nodes. Specific to integration of audio/visual transmission, there remains the future possibility of integrating a customizable FPGA design with the MIPI alliance M-PHY interface. The M-PHY protocol is a physical layer adaptation specifically designed for high throughput, low powered wireless applications that is currently utilized in cell phones [71,72,73] and has the potential to be applied to IoT edge nodes for wireless applications.

There is always room to improve with regard to lowering latency and increasing throughput, while the current state-of-the-art FPGA NICs are reaching throughputs of 100 Gbps, the Ethernet Roadmap by the Ethernet Alliance suggests that current maximum throughput requirements are seeing demands of 400 Gbps to 1 Tbps [74]. That demand leaves a significant room for improvement over today’s designs.

## 4. Mid-Range IoT Solutions

The majority of IoT devices will fall under the mid-range category, which we define as nodes that focus on balancing power saving features with other application specific requirements, such as range extension [75,76], limiting form factor [77], or utilizing neural networks [78,79]. The primary difference between these solutions and those in the high power category is that mid-range IoT implementations must take power usage into account, as constant power sources are often not available, and therefore energy saving techniques must be used to prolong battery life. The advances presented within this section, while most applicable to mid-range solutions, can also be applied to high-performance IoT implementations if necessary.

### 4.1. Data Management

Data management in power limited systems poses unique obstacles that are not present within other types of networks. For example, devices will often go into sleep mode, which may result in lapses of communication or lost data. Further, data caching within CoAP and HTTP stacks have limitations unique to IoT applications, as clients will need explicitly forward or reverse proxy nodes that are meant to utilize caching. The network dynamics of IoT edge nodes mean that some nodes may either go out of range or otherwise may not be immediately available for remote communication, leading to the loss of inter-node communication. Edge nodes and proxies need to be able to adjust to these ever changing states of nodes.

Data persistence is a necessity within IoT networks, as lost packets must have a way to be recovered, especially in systems where edge nodes are frequently unavailable due to being out of range or in a sleep state. An FPGA middleware server was designed for this purpose, which allows for persistent data within a network [80]. This middleware server acts as a mediator between a server and edge nodes, decoupling the server from the critical data path: when a node sends a request to the server, it is intercepted by the middleware, which then sends an ACK signal back to the node. This allows the client device to continue its operations without waiting for an ACK from the server, which may have a longer response time than the middleware server due to distance. The middleware stores data packets in persistent memory, allowing them to be retrieved from the client nodes or server in the case of dropped packets or data corruption.

Another approach for maximizing efficiency of IoT edge nodes uses remote direct memory access (RDMA) between nodes, dubbed Smart Remote Memory (StRoM) [81]. StRoM sees direct integration between the NIC and the direct memory of a node. This facilitates data handling between nodes without requiring the CPU to act in the process, resulting in reduced power consumption. A series of Remote Procedure Calls (RPCs) were implemented that allow for a variety of instructions to operate on the remote memory. This can be valuable when single nodes exist only to retrieve data from sources such as thermometers or humidity sensors, with requirements that their data be remotely accessed by external server calls [82,83]. Additionally, this grants the ability for an MCU on a node to go into sleep mode, while its memory can still be accessed so long as the NIC remains in an active state.

### 4.2. Network Security

Networking security within IoT can also prove to be a challenge, with unique requirements for each system. Traditional IP security, such as Transport Layer Security (TLS) [84] and Datagram Transport Layer Security (DTLS) [85], do not fit into the typical IoT paradigm. For example, these two protocols require multiple rounds of secure hand shaking (increasing overhead), mandating that both ends of a secure channel maintain their respective states until the channel is closed. Further, TLS and DTLS do not guarantee the security of application level data. For this reason, data must be secured prior to being transmitted from the application to the network layer.

For means of data security within an IoT network, a Lorenz IP core was implemented to create a chaotic solution for low-power data encryption [86]. A chaotic system is one that produces seemingly random results from an outside perspective, but are predictable if the starting conditions are known. The Lorenz system, specifically, is a set of ordinary differential Equations (ODEs) that can be used to produce these chaotic results. For implementing ODEs within an FPGA, this design utilized the Runge–Kutta (RK) approximation method for results. The RK method is an iterative method for approximating solutions of simultaneous nonlinear equations. For the initial conditions, which determine the iterative outputs of the ODEs, the receiver and transmitter both had hard-coded values so that the receiver could decode the received values, while the Lorenz/RK combination is a unique solution for low power data encryption, the hard-coded initial value poses a significant security risk. Should the bit stream be compromised via extraction, the hard-coded value could easily be determined, breaking the encryption system [87,88,89].

Equally important as the concept of data integrity is the idea of network intrusion detection. Network intrusion, or the unauthorized activate on a network, can lead to the compromise of not just data, but entire nodes and systems. A low power binary neural network (BNN) was developed for the detection of network intrusion with an 82% accuracy at a cost of 1.5 W [90]. This implementation analyzes network packets for fuzzers, backdoor attacks, denial of service attacks, reconnaissance attacks, and shellcode/worms. The primary improvement for this approach would be towards training a more accurate network, with a success rate of greater than 90%.

### 4.3. Data Transmission

IoT networking applications could, and still can, benefit in areas that are outside of security and data management. Recent advancements have utilized the powerful feature of FPGAs for remote reconfiguration, which enables on-the-fly modification of configuration bit streams. In addition, new innovations look to managing service migrations and unifying communication busses, all of which are explored within this subsection.

A custom Software Defined Radio Network (SDR), tinySDR, implemented a unique approach to integrating a long range (LoRa) physical interface [15]. On top of allowing for networking communication in the range of multiple kilometers, LoRa implemented a method for remote reconfiguration of an FPGA with an integrated MCU. We focus specifically on the reconfiguration method and leave the exploration of the LoRa PHY up to the reader. When a device reconfiguration command is detected by the MCU via the MAC layer, the FPGA is turned off. The compressed data, sent over the LoRa PHY, is decompressed by the MCU and loaded into the configuration flash for the FPGA in 30 kB packets. Once the entire configuration bit stream has been decompressed and stored in flash memory, the FPGA is rebooted.

The primary area for improvement in this design exists in the ability to utilize the FPGA’s dynamic reconfiguration property. Assuming that the incoming reconfiguration data is a minor improvement, as opposed to an entire design change, the FPGA should not need to be entirely reflashed. With this assumption, dynamic reconfiguration could be used to implement the benefits described in Section 5.3.

Another focus of recent contributions to IoT networks relying on FPGAs include migrating services between servers and endpoints for either maintaining network latency requirements or increasing network ranges. This requires IoT nodes to have modifiable IP addressing schemes as a service transverses the network nodes, requiring special protocols such as Open Flow. In order to simplify this task, a recent work aims to implement service migration by relying on network transparency, which is when a networking protocol transmits data over a network in a way that the transfer process is invisible to the application layer [16]. This is crucial for service migration, as resource constrained IoT nodes can entirely disregard the networking protocol while still allowing for service migration. Further, the work maintains network consistency, which facilitates lossless packet transfer without additional hardware being implemented into the system.

The work implemented by [16] relies on reconfigurable partitions within nodes, allowing exactly one service per partition. By utilizing dynamic partial reconfiguration, the node can have entirely different services, so long as the configuration is locked to designated partitions within the FPGA. This is a unique implementation of partial reconfiguration, and has the potential to be combined with the reconfiguration principles introduced in [15] for configuration updates.

The final work explored within this paper aims to unify common communication protocols found throughout IoT networking [91] with an adaptation of a mixed media network. This approach, aiming to unify serial transmission methods, was implemented on satellite systems, and provided support for SPI, UART, RMII, RGMII, and SGMII. The FPGA was implemented as a transceiver between each serial link, and provided the translation to send the received data over Ethernet to other links for reception. The low speed nodes, SPI and UART, were limited to data rates of 0.1 to 10 Mbps, whereas the high speed nodes like RMII, RGMII, and SGMII are capable of reaching rates up to 1 Gbps. The mixed media switch implementation shows power consumption rates of 200–700 mW, allowing for low power applications so long as an Ethernet connection is available. Future adaptations, especially for IoT purposes, may want to look towards implementing the mixed media bus onto a wireless network.

### 4.4. Future Directions

Even with the previously discussed improvements in IoT networking, there still remains significant potential for networking improvements through the utilization of FPGAs outside the improvements listed above. Specifically, we should look towards data recovery and persistence without relying on middle hardware. The implementation of middle hardware increases the overall system design complexity and power consumption. Future adaptations should look at custom, low-overhead header implementations for improving data persistence capabilities.

Further, FPGAs can also be utilized to support nodes in sleep states. There exist off-the-shelf, ultra-low powered FPGAs that can remain in an awake state while the MCU goes into a sleep state. The FPGA could be used to wake the MCU in the case of a design-specified event or an incoming network request. This can allow for nodes to remain in a sleep state indefinitely, removing the need for the node to routinely wake up and check the status of its network/environment.

Finally, it must be mentioned that there will always be a demand for lower power designs, and current designs can utilize new FPGA technologies, such as smaller manufacturing processes, to reduce power consumption. We expect mid-range IoT applications to expand, as improvements continue for both FPGA designs and device manufacturing. There will always be a demand for less-power hungry designs than those that are currently available. There are instances, especially when reducing power consumption is paramount, that an ASIC may be a better choice for a design rather than an FPGA. If a design can suffice without the reconfigurability, low non-recurring engineering costs, and shorter development times offered by FPGAs, then a custom ASIC may prove to be to a better alternative. For more information on ASICs in IoT networks, we encourage the reader to refer to references [92,93,94].

## 5. FPGA Optimization Techniques

We summarize the primary techniques used within the reviewed papers and describe how they may be carried forward into new works. Further, because of the similarity of designs in Section 3.3, we directly compare them against each other. Since the other subsections do not share the same similarity of their respective works, we only present the benefits and drawbacks of their approaches.

### 5.1. Latency Reduction

Latency measurements, and what is considered to be "low latency," is application specific. As exemplified by the two works covering latency improvement within this paper, a low latency application could see response times as low as a few hundred nanoseconds, or as high as a few milliseconds. Regarding audio/visual transmissions, focusing on latency reduction comes in forms of highly-efficient codecs that can compress and decompress transmitted video signals. By moving codecs from the application layer in software closer to the networking layer in hardware for application specific designs, we can observe significant latency reductions in video transmissions. For example, in [50], TICO video compression was used over a fiber link, where the encoder and decoder were implemented on a Xilinx FPGA in conjunction with Block RAM (BRAM) to store incoming packets.

Data-specific applications that do not revolve around audio/visual transmissions can observe similar design flows, where data comprehension is moved from the application layer closer to the network layer. By integrating multi-gigabit IPs from vendors such as Intel and Xilinx, multiple layers of data abstraction can be removed depending on design requirements, and TCP/IP packets can be directly read and manipulated in hardware, thereby reducing computation times. For example, the work in [49] moves financial trading services to a custom FPGA design that allows for user-defined trades to be executed with low latency. The extracted data from incoming Ethernet packets are compared against purchase and sell requests from the user, and the corresponding purchase/sell orders are subsequently sent back to the server when a match is detected.

### 5.2. Throughput Improvements

While current ASICs support high levels of throughput, they do not offer the same level of configurability that is available on FPGAs. This is a necessity for reasons mentioned in the previous subsection: moving application level services, such as video decoding or financial trading, to hardware can significantly reduce latency and improve power and resource efficiency. The three high-throughput papers covered within this review implement their own individual network stacks with a direct interface to an Ethernet PHY. Two of the works, Limago and EasyNet, were implemented using High Level Synthesis (HLS), which allows designers to modify the work without interacting with any register transfer level (RTL) logic. However, being solely developed in HDL has its advantages as well. For instance, Corundum is able to minimize resource usage compared to EasyNet and Limago, as the lack of specificity in HLS leads to extraneous resource usage. The resource usage in each work is compared in Table 1.

The devices compared within Table 1 are all from the Xilinx Virtex Ultrascale+ family of FGPA devices, which is based on a 14 nm–16 nm FinFET architecture. While the devices outlined in the table differ in performance capabilities, the similarity in architecture allows for comparable resource usage between devices. We chose the Xilinx family of devices for comparison between the three designs, as each work provided synthesis results for the Xilinx Virtex Ultrascale+ family, allowing for the most direct comparison between design implementations. For resource comparison, we observed the Look Up Table (LUT) utilization, Flip Flop (FF) utilizaion, Digital Signal Processor (DSP) utilization, and Block Random Access Memory (BRAM) utilization.

While the developers of EasyNet did not list the amount of FF required for their design, the other metrics are still compared. It should also be noted that these designs are listed for a general comparison, as the resource usage will vary slightly between different models of FPGAs. Resource usage can be affected by compiler settings, timing constraints, resource availability on a device, as well as available routing.

In Table 1, Limago is listed on the VCU118 board with a Virtex Ultrascale+ FPGA and is configured without the aforementioned Window Scaling option and optimized for 10k total connections. EasyNet is listed as being configured for a U280 FPGA from Xilinx, and Corundum is listed as being optimized for a Virtex Ultrascale+ VU3P-2 FPGA with an ADM-PCIE-9V3 add-in card.

The benefit of using specialized HDL is clear from Table 1: Corundum resource usage is approximately half of either EasyNet or Limago in all categories except for the BRAM usage. This will allow for Corundum to be available for smaller FPGAs, as well as allowing it to work in tandem with larger HDL designs on the same FPGA that may need the resources for custom, application-specific purposes. Further, by reducing the required resources, the design will see a reduced power consumption.

### 5.3. Partial Reconfiguration

Partial reconfiguration updates a portion of an FPGA design that is to be reconfigured while allowing for the rest of the design to continue functioning. The implementation for partial reconfiguration differs between FPGA vendors, but the principles remain the same. Variants of logic that can be utilized to reconfigure portions of the FPGA are called personas, where exactly one persona can occupy a physical portion of the FPGA at a time. Partial reconfiguration can be applied to designs that may have multiple permutations that do not operate simultaneously and designs that require continuous operation. There are multiple options for integrating partial reconfiguration, such as over a PCIe interface, through custom FPGA firmware reading from memory, using a controller on either a hard-core or soft-core processor, or, in the case of tinySDR [15], over a LoRa.

Partial reconfiguration, a feature unique to FPGAs, provides an abundance of benefits for designs that utilize it. One of the main benefits is cost reduction: hardware resources can be reused by individual personas, as opposed to having replicated hardware for designs that run all personas simultaneously. This results in the ability for a smaller FPGA footprint and power reduction, both of which are necessities in mid-range IoT applications. In addition, there are increased uptimes, as updates can be dynamic, allowing for the design to continue running so long as the updated portion is within a reconfigurable persona [95,96].

## 6. Conclusions

There exist advancements in IoT networking, as shown in the previous sections, that rely heavily on the capabilities of FPGAs. However, not all applications will be suited for the use of FPGAs, and may instead benefit from utilizing pre-fabricated ASICs or embedded processors. In many instances, simple tasks, such as those handled in the application layer, may experience a faster development period if the design were to be implemented on embedded processors as opposed to trying to integrate functionality on devices as complex as FPGAs. Similarly, complex hardware integration services requiring little-to-no customization may see a financial benefit to utilizing already available ASICs. ASICs will typically allow for lower power usage than their FPGA counterparts, and can typically come in a smaller package when compared against a similar design implemented on an FPGA. Additionally, without the operating requirements of relying on LUTs, and instead using application specific logic, ASICs can achieve higher operating frequencies than FPGAs, although, this may not be beneficial in medium-range IoT networks where power saving is paramount.

Despite the FPGA device not excelling in these categories, FPGAs still have a significant place in the future advancements of IoT networking. If a design were to require a network PHY interface with customizable or unique features otherwise not implemented on an already existing ASIC, such as the novel approaches discussed within this paper, then FPGAs will remain the best candidate for the job. The ability to quickly reconfigure FPGAs allows for the correction of design bugs and implementation of new features without the overhead that comes with ASICs allows for FPGAs to be at the forefront of IoT networking advances.

The ability for dynamic reconfiguration will also enable approaches to handle current gaps in networking technology. Unique to FPGAs, dynamic reconfiguration will allow for on-the-fly bug fixes and service updates that are not realistic on ASICs or other types of processors. Further, with open source works, such as some of those listed in previous sections, it is possible to easily build on and combine previous designs onto one FPGA to fit the unique needs of each IoT network. Furthermore, there are many options with varying costs for ultra-low power FPGAs. These can, and should, be used for future IoT networking designs as power saving features continue to be a priority for IoT applications. FPGAs significance in IoT networking innovations can be summarized as this: IoT implementations are often tailored to their particular application, and, as such, will have unique networking requirements. FPGAs provide the ability to implement these requirements in both a cost and time efficient manner.

Within this paper, we reviewed recent advancements in FPGA and IoT networking and discussed their contribution to the field. We also proposed areas for future research, such as improving throughput and latency times, decreasing power consumption, working with data packet recovery, and implementing additional features for lower-power sleep modes.

## Figures and Tables

**Table 1:** Resource usage from Limago, EasyNet, and Corundum. N.L.: Not Listed.

System Name	FPGA	LUT Usage	FF Usage	DSP Usage	BRAM Usage
Limago	VCU118	120k	178k	N.L.	441
EasyNet	U280	141k	N.L.	9	522
Corundum	XCVU3P	62k	74k	N.L.	331

## Data Availability

Not applicable.

## Abbreviations

The following abbreviations are used in this manuscript:

FPGA	Field-Programmable Gate Array
Gbps	Gigabit per second
PHY	Physical Layer
Tbps	Terabits per second
OSI	Open Systems Interconnection
PDU	Protocol Data Units
HLS	High Level Synthesis
HDL	Hardware Description Language
CAT5	Category 5
CAT6	Catagory 6
LFN	Long Fat Pipe Network
AXI	Advanced Extensible Interface
ARP	Address Resolution Protocol
MAC	Media Access Control
RX	Receive
TX	Transmit
SAR	Segmentation and Reassembly
IDE	Integrated Development Environment
RTL	Register Transfer Level
TCP	Transmission Control Protocol
IP	Internet Protocol
FIFO	First In, First Out
NIC	Network Interface Card
IPv6	Internet Protocol Version 6
IPv4	Internet Protocol Version 4
CoAP	Constrained Application Protocol
MQTT	Message Queue Telemetry Transport
6LoWPAN	IPv6 Over Low Power Wireless Personal Area Networks
